# Recent Advances in the Therapeutic Efficacy of Artesunate

**DOI:** 10.3390/pharmaceutics14030504

**Published:** 2022-02-25

**Authors:** Ngonidzashe Ruwizhi, Rejoice Bethusile Maseko, Blessing Atim Aderibigbe

**Affiliations:** 1 Department of Chemistry, University of Fort Hare, Alice Campus, Alice, Eastern Cape 5700, South Africa; 201515559@ufh.ac.za; 2 Department of Chemistry, School of Science and Technology, Sefako Makgatho Health Sciences University, Ga-Rankuwa 0208, South Africa

**Keywords:** artesunate, malaria, anticancer, tumour, antiviral, COVID-19, skin diseases

## Abstract

Artesunate, a semisynthetic artemisinin derivative, is well-known and used as the first-line drug for treating malaria. Apart from treating malaria, artesunate has also been found to have biological activity against a variety of cancers and viruses. It also exhibits antidiabetic, anti-inflammatory, anti-atherosclerosis, immunosuppressive activities, etc. During its administration, artesunate can be loaded in liposomes, alone or in combination with other therapeutic agents. Administration routes include intragastrical, intravenous, oral, and parenteral. The biological activity of artesunate is based on its ability to regulate some biological pathways. This manuscript reports a critical review of the recent advances in the therapeutic efficacy of artesunate.

## 1. Introduction

**Figure 1:**
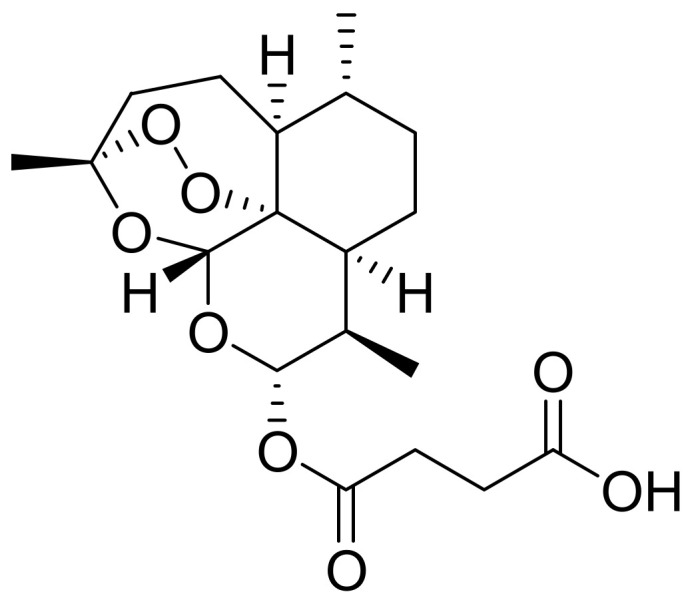
Structure of Artesunate.

Artesunate (1) (ART), also known as dihydroartemisinin-12-α-succinate, is a semisynthetic peroxide-bridged sesquiterpene lactone compound (Figure 1) [1] derived from artemisinin, the bioactive component of the Chinese medicinal herb called *Artemisia annua* [2]. The production of ART from artemisinin involves a two-step reaction of reduction and esterification using diisobutylaluminum hydride (DIBAL) and succinic anhydride, respectively [3]. Alone, ART is the first-line drug for the treatment of severe malaria but is also used combined with another drug for treating non-severe malaria [4].

The presence of a 1,2,4-trioxane core incorporating an endoperoxide linkage is very important for its activity [5]. ART has better absorption, solubility, and pharmacokinetics than artemisinin. Its administration can be intramuscular, oral, rectal, and intravenous [3]. When orally administered, ART has a short half-life ranging from 20 to 45 min, during which it is metabolized, through esterase-catalysed hydrolysis, to dihydroartemisinin, the active metabolite responsible for the antimalarial activity of artesunate [6].

The short half-life results in less stability which consequently lowers its bioavailability, pharmacokinetics, and pharmacological activity. The short half-life of ART also necessitates the need to repeat administration for absolute cure, which can cause drug resistance [7]. Over the last two decades, studies have shown that ART is not only effective against malaria but also has some anticancer effects on various tumour cell lines, both in vitro and in vivo, [8], plays certain roles in treating lupus [9], has efficacy against several viruses [10,11], and exhibits antidiabetic properties [12].

Artesunate displays antitumor activity, and its effect on non-apoptotic cell death includes the following mechanisms: autophagy, oncosis and ferroptosis [13,14,15,16]. Artesunate also affects other multiple hallmark events of cancer development by inhibiting cancer cell proliferation and invasion, inducing cell cycle arrest, disrupting cancer signalling pathway, causing oxidative damage, and inducing cell apoptosis. It also acts by inhibiting angiogenesis and as a metastasis agent [17,18]. The trimeric ART derivative, TF27, showed that ART interacts with human cytomegalovirus (HCMV) through exportins and mitochondrial and NF-κB pathways [19,20]. Furthermore, ART has been reported to have anti-atherosclerosis [21], anti-inflammatory, and immunosuppressive activities [22,23]. This review article focuses on the therapeutic efficacy of artesunate.

## 2. Malaria and Its Parasite Life Cycle

Malaria is a parasitic vector-borne disease caused by *Plasmodium* spp. transmitted by female *Anopheles* mosquitoes [24]. Although malaria is life-threatening, early diagnosis and appropriate treatment lead to it having an excellent prognosis [25]. Global data show that around 229 million people are affected by malaria, while about 409,000 succumb to it annually; hence, efforts are needed to prevent and control it [26]. Children under the age of 5 years and pregnant women are at a higher risk of being attacked by malaria and have a greater likelihood of developing complications [27]. Early treatment of malaria reduces mortality to 10–20%, while untreated severe malaria has a 100% mortality [28]. Despite all the major efforts to control and eradicate it, malaria remains a devastating disease in endemic regions [29]. Artesunate is well known for its biological efficacy in treating both uncomplicated and severe malaria [4,30].

**Figure 2:**
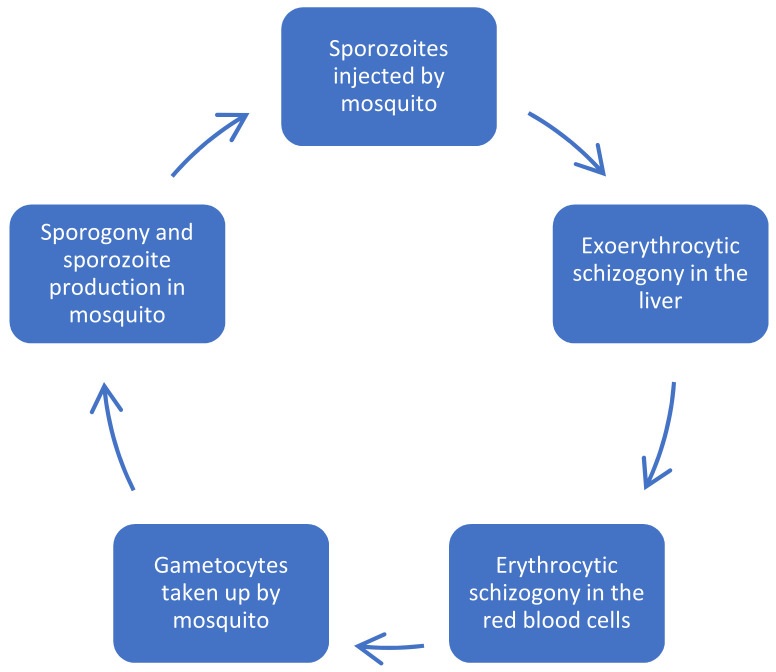
Schematic life cycle of *Plasmodium falciparum*.

Understanding the life cycle of the malaria parasite is required in the successful development of effective antimalarials (Figure 2). The female *Anopheles* mosquito develops its eggs from the proteins obtained upon feeding on human blood [31]. As it feeds, infective sporozoites are inoculated into the bloodstream and circulate until they invade the hepatocytes, where they replicate for 7–14 days. The pre-erythrocytic stage, under which the incubation period falls, shows no visible symptoms. After the pre-erythrocytic stage is the erythrocytic stage where the parasite emerges as merozoites from the liver. Invasion of the red blood cells (RBCs) by merozoites leads to their multiplication into an erythrocytic schizont, which releases more merozoites upon bursting. More RBCs are invaded upon release of merozoites, and thereby lengthening the blood stage of the malaria life cycle [32].

Some of the merozoites develop into gametocytes, and for these gametocytes to be transmitted to the host, they will need to be absorbed by another mosquito. The gametocytes complete their sexual reproduction stage when absorbed by another mosquito. This results in the ending of the mosquito cycle, which takes about 9–14 days. Finally, the sporozoites migrate to the salivary glands, ready for being inoculated into another bloodstream when the mosquito gets another blood meal, thus completing the malaria life cycle [33]. *P. ovale* and *P. vivax* have different life cycles because some of the sporozoites do not multiply in the liver stage and develop into schizonts, but are rather concealed within the liver becoming hypnozoites which cause relapse years later after the time of first infection [34,35].

Among the many species of Plasmodium, *P. falciparum* is unique because it invades any RBC and can cause several infections on one RBC, thus causing fast multiplication, which quickly worsens the disease. Structural and functional changes such as sequestration, inflammation, and endothelial dysfunction happen to RBCs, and these lead to severe malaria [35].

### 2.1. Mechanism of Action of Artesunate on Malaria and Its Structure–Activity Relationship

In the body, ART gets converted to dihydroartemisinin, which has a higher half-life of around 45 min. Mechanisms of action for ART (like any other artemisinin) include inhibiting heme polymerization, generating ROS, destabilizing parasite membrane, alkylating proteins and inhibiting PfATP6 [6].

The endoperoxide moiety of artesunate generates ROS which helps during its mechanism of action. Other mechanisms include inducing apoptosis and cell cycle arrest and inhibiting tumour angiogenesis [36]. There is a sequence of events that lead to the fatal damage of the parasite when exposed to artesunate. Iron-induced reduction inside the malaria parasite activates the endoperoxide linkage, which in turn triggers the release of several reactive intermediates. These include high-valent iron-oxo intermediates, cytotoxic carbon-centred free radicals, and electrophilic alkylating agents, and all these ultimately damage the parasite to death [37,38].

ART binds to hemin forming a hemin–artesunate adduct. This adduct prevents hemozoin formation leading to heme accumulation which is toxic to the parasite [6]. Heme accumulation induces carbon-centred free radicals generation, which in turn alkylate heme and cysteine proteases, such as falcipain in *P. falciparum*, causing oxidative damage to the parasite membrane, and ultimately its death [38,39].

The parasite retains hemozoin, a storage form of hemin, after digestion of haemoglobin. ART has a high affinity for hemozoin, and this results in the drug selectively accumulating by the parasite [39]. ART is selective towards parasite-infected erythrocytes over normal ones because of the iron-dependent activation of the endoperoxide bridge [38].

### 2.2. The Efficacy of ART-Based Formulations on Malaria

Parenteral ART administration is preferred for the treatment of severe and cerebral malaria [30]. Agbo et al. prepared nanostructured lipid carriers (NLCs) for the intranasal delivery of ART. Solidified reverse micellar solutions-based lipid matrices were employed to encapsulate the hydrophobic ART in NLCs. In vivo antimalarial studies on mice revealed that one of the ART-loaded NLCs reduced parasitaemia (54.70%) in infected mice, and the results were comparable with those obtained through intramuscular administration (58.80%) [28].

Antimalarial activity for the intranasal administered ART-loaded NLCs was 33.28% comparable with 42.18% for the intramuscular one. The NLC formulation exhibited a higher antimalarial activity than pure ART, suggesting that intranasally administered ART-loaded NLCs can be used to enhance the antimalarial activity of ART, in addition to them being safe, convenient, and effective [28,40].

Marco-Hernandez et al. reported a diagnostic challenge with the intravenous treatment of *P. falciparum* malaria on a patient with splenectomy. The patient was initially identified to have severe malaria (parasitaemia 4.7%) and acute kidney injury, with creatinine level at 1.3 mg/mL. Although intravenous ART (2.4 mg/kg) was started immediately after diagnosis, the blood smear 24 h post-ART showed parasitaemia increase of up to 8.7% [41].

Suspected artemisinin resistance led to the initiation of endovenous quinine (10 mg/kg three times a day) plus doxycycline (100 mg twice a day) three days later, but the sequencing of *kelch13*-propeller domain revealed no artemisinin resistance markers. Revaluation of malaria smears revealed pyknotic forms leading to deductions that non-viable malaria parasites could not be cleared from the bloodstream due to the patient’s splenectomy. A negative blood smear was finally obtained 28 days after treatment [41].

Driscolli et al. reported drug-induced autoimmune haemolytic anaemia following ART treatment for malaria. Although the patient showed a good response to ART, symptomatic anaemia (Hb of 64 g/L) and haemolysis were noted 16 days post-treatment. Both direct antiglobulin and antibody screen was positive. Two weeks after commencing prednisolone (1 mg/kg) therapy, treatment success was noted with complete normalisation of Hb and rapid haemolysis resolution. Though cases that have a positive direct antiglobulin test are rare, their increase in reports raises a concern that ART may cause drug-induced autoimmune haemolytic anaemia [42].

Mahdi et al. reported that ART treatment of severe *P. falciparum* malaria can induce late acute pancreatitis. The patient was intravenously administered five doses of ART (2.4 mg/kg). The patient’s biofilms revealed no malaria parasites 72 h post-treatment. On day 8 after admission (5 days after testing negative to blood parasite), the patient showed pancreatitis, although having no previous risk factors for pancreatitis. The pancreatitis was seen as a possible adverse reaction to intravenous ART [43] after using the adverse drug reaction probability score. Intravenous fluid and pain management led to a rapid response of pancreatitis, and it did not reoccur even up to 2 months post-treatment [25].

Severe malaria anaemia (SMA) usually results in Hb levels decreasing to dangerous levels, and this is usually worsened when ART is used as the only treatment since it does not increase Hb level but in effect mildly lowers it [44,45]. Siewe and Friedman designed a mathematical model that helps in increasing Hb level in SMA while controlling parasitaemia. Malaria parasites secrete plasmodium macrophage migration inhibitory factor (PMIF) which suppresses RBCs recruitment, thereby decreasing Hb level in the blood [24].

Simulations using artesunate, known for primarily decreasing parasitaemia, and epoxyazadiradione, an anticancer drug, showed complementary results. While the artesunate decreased parasitaemia, epoxyazadiradione increased Hb level by acting against PMIF. The model can also be used in the treatment of other parasitic diseases that cause anaemia and where MIF plays a crucial role [24].

Ismail et al. prepared novel dimeric ART-glycerophosphorylcholine (Di-ART-GPC) liposomes for the treatment of *P. falciparum* malaria. In vitro antimalarial activity evaluation against *P. falciparum* i-RBCs showed significant growth inhibition of 3D7 strain, with Di-ART-GPC liposomes and conjugate exhibiting IC_50_ values of 0.39 and 1.90 nM, respectively. These inhibition values showed more potency than the ones of parent artesunate (IC_50_ = 5.17 nm) and ART-loaded liposomes (IC_50_ = 3.13 nM). In vivo antimalarial results showed that Di-ART-GPC liposomes have a 2- to 3-fold higher antimalarial activity at a low dosage of 15 mg/kg compared with free ART. Improved life expectancy and delayed parasitaemia recrudescence in the mice, together with the other aforementioned results, showed that ART efficacy can be improved by using Di-ART-GPC liposomes [3].

Pregnant women who contract malaria experience reduced birth weight, while children below 5 years of age experience poor growth and mental retardation [46]. Shehu et al. evaluated the effects of prenatal exposure to ART on the cerebral cortex development in Wistar rat foetuses. The ART was intragastrically administered on gestation day 6 with safe or high human doses of 8 mg/kg or 16 mg/kg (4-2-2 or 8-4-4, respectively) or three days. Postnatal morphological evaluations showed significantly stunted growth of the tail, limbs, and decreased brain cerebral morphometry while the high dose induced severe embryo loss [27].

Histological results showed decreased cell proliferation and delayed pyramid cells maturation on rats on the safe dose compared with the controls. Complete retardation of cerebral development was noted on the pups administered with a high dose. When the neurons specific for calbindin D28K, a calcium-binding protein, were immunolabelled, the pyramid cells in ART-treated pups showed fewer positive cells with less staining compared with the control. The results indicated the narrow histological and neurological safety margin of ART [27].

Several research reports have revealed that some other antiplasmodial drugs can work synergistically with ART. These include combinations such as pyronaridine–ART and ART–amodiaquine, which has been found to display enhanced efficacy [47,48]. Mina et al. reported that ART interacts synergistically with 4-chloro eugenol (4CE), a potent antiplasmodial drug with activity ranging between 1.5 and 5 µM against drug-resistant *P. falciparum*. In vivo antiplasmodial activity using Swiss albino mice infected with *P. yoelli nigeriensis* showed that a combination of ART and 4CE had chemo-suppression of 91.4% compared with 55% and 47% for ART (9.5 mg/kg) and 4CE (88 mg/kg), respectively (Table 1). The results depicted below showed dose-dependency (Table 1) [26].

The mean survival time for the mice administered ART + 4CE was longer (16.3 days) compared with those administered the individual drugs. Oral dosage of the drugs was conducted for cerebral malaria studies by noting Evan’s blue concentration visible in brain tissue. The combination (2×ED50 of ART + 4CE) reduced blood–brain integrity loss (4.73 µg/g of Evan blue) compared with the individual drugs which had 20.99 µg/g and 17.98 µg/g of Evan’s blue visible for 4CE (166 mg/kg) and ART (20 mg/kg), respectively. The results showed that 4CE can synergistically interact with ART to induce oxidative stress against drug-resistant *P. falciparum* [26].

In addition to its antimalarial activity, heparin is also used as a nanocarrier because of its biocompatibility and biodegradability [49]. Research has reported that malarial parasites use the cellular surface receptor, heparin sulfate, for initial binding and recognition [50]. Ismail et al. developed ART–heparin (ART–HEP) conjugate-based nanocapsules for intracellular release of ART in malaria treatment. In vitro antimalarial activity of the ART–HEP nanocapsules on *P. falciparum* 3D7 strain showed that free ART (IC_50_ = 6.27 nM) exhibited a higher inhibitory effect than the polymer nanocapsules (IC_50_ = 10.16 nM). This might have been due to ART being gradually released from the nanocapsules. Pharmacokinetic studies showed that the ART–HEP nanocapsules had higher blood circulation than free ART due to shielding by the outer heparin. A slightly higher peak plasma concentration (C_max_ = 18.12 µg/mL) was exhibited by ART–HEP nanocapsules compared with that of plain ART of 14.13 µg/mL. The higher residence time and elimination rate, together with declined plasma clearance (Table 2), make ART–HEP nanocapsules a potential ART carrier in malaria chemotherapy [7].

Kone et al. studied the clearance times of *P. falciparum* using light microscopy and quantitative polymerase chain reaction (qPCR) in 221 volunteers from two Malian villages (121 from Faladje and 100 from Bougoula-Hameau) after ART monotherapy. The patients were observed for 28 days, and their blood smears and spots were collected for qPCR and microscopy. ART PCR-corrected cure rate was brought to 100% in both villages. Parasite clearance assessment by microscopy showed that a median time of 40 h was needed to clear all parasitaemia in Faladje compared with 32 h of Bougoula-Hemeau [29].

qPCR results 72 h post ART treatment showed that 68.5% of the patients had residual parasitaemia and a mean residual parasitaemia of 2.9 for Faladje (54) samples. Bougoula-Hameau (50 samples) had 40% of patients with residual parasitaemia and 0.08 mean residual parasitaemia. Drug resistance molecular markers revealed one *Pfdhfr–Pfdhps* quintuple mutant and a non-synonymous *PfK13* mutation in Bougoula-Hameau only. Though ART treatment was effective, the prolonged parasite clearance may be an early sign of the development of *P. falciparum* resistance to ART in the analysed individuals [29].

There have been several reports on severe delayed autoimmune haemolytic anaemia post ART treatment [51]. This usually happens when the patient has a weakened immune system due to severe malaria, human immunodeficiency virus, etc. [52]. Rabaneda-Gutierrez et al. reported haemolytic anaemia following ART treatment for severe malaria in paediatric patients (6-year-old boy, 6- and 4-year-old girls). The two girls had parasitaemia of 5% and 15%, respectively, and received intravenous ART (2.4 mg/kg/dose for every 12 h for two doses and 24 h after that) followed by piperaquine–artenimol for 3 days. Parasitaemia disappeared 48 h after treatment initiation. Laboratory test 10 days after treatment initiation showed possible haemolytic anaemia with a drop in haemoglobin from the one recorded 5 days before (from 9 g/dL to 7.4 mg/dL in 6-year-old and from 8.8 g/dL to 6.3 mg/dL in the 4-year-old) [53].

Lactate dehydrogenase (LDH) increased from 674 U/L to 751 U/L in the older and from 738 U/L to 1831 U/L in the younger girl. The same haemolytic anaemia findings were shown by the boy after administration of the same treatment. He had a 2 g/dl drop in haemoglobin, hyperbilirubinaemia of 2.08 mg/dL from 1.5 mg/dL previously, and LDH elevation from 354 IU/L to 1831 IU/L. Prednisolone was administered at 1 mg/kg/day for 3 days, and the lab tests showed favourable results. These findings showed that paediatric patients are at potential risk of haemolytic anaemia following intravenous ART [53].

Landre et al. reported a case of a patient who was admitted into the ICU with severe malaria, having a medical history of hypertension, diabetes, and obesity. Blood smears showed positive *P. falciparum* (675,000 parasites/µL), confirming severe malaria with biological parameters showing acute kidney injury, respiratory and cardiovascular failure, and hyper-parasitaemia. Intravenous ART (2.4 mg/kg) was started from day one up to 7 days. Continuous venovenous haemodialysis was carried out for the first 3 days, and norepinephrine was used as support for 10 days [54].

The patient was discharged from the ICU 4 weeks after admission with a better condition but presented with cough, hyperthermia, and pulmonary opacity. He presented haemolysis, low platelets, and undetectable haptoglobin. A positive *P. falciparum* (6,240,000 parasites/µL) test was confirmed, and this led to the diagnosis of severe malaria recrudescence. Second ART treatment was initiated for 3 days, followed by intravenous quinine (780 mg/8 h) for 5 days and oral artemether–lumefantrine for 3 days, leading to successful treatment [54]. The recrudescence of hyper-parasitaemia might have been caused by either being infected with at least two *P. falciparum* clones or the other underlying conditions, such as obesity, diabetes, and hypertension [55].

The combination of ART and amodiaquine is known for its efficacy in treating uncomplicated malaria and is recommended by the World Health Organisation [56]. Mohammed et al. assessed the efficacy of ART–amodiaquine (ART + AQ) as the first-line treatment for uncomplicated *P. falciparum* malaria in Eritrea. A total of 103 patients (65 males and 38 females) diagnosed with malaria were enrolled in the study for 28 days, with asexual parasite density at a geometric mean of 7553 parasites/μL at recruitment. On day 3, there was rapid parasitaemia clearance, with only 3/102 cases having asexual parasites detectable in blood film (Table 3). A total of 99 patients completed the analysis since 1 withdrew and 3 were lost to follow-up. All the recurrent parasitaemia was assumed to be due to treatment failure, not re-infection because the results were not PCR-corrected. The fact that only three cases had parasitaemia on day 3 means that the ART component is still very effective because of its effectiveness in initial parasite clearance and suggests no resistance to ART [57,58].

Zodda et al. reported a case of a previously healthy 33-year-old man who was admitted with severe malaria and cerebral parasitaemia. Laboratory results on complete blood count produced a haemoglobin level of 10.2 g/dL, white blood cell count of 12 k/µL, haematocrit of 32%, and 13 k/µL platelet count. Hepatorenal impairment was also noticed, and blood smears showed malaria parasites within 42% of RBCs. The patient was first administered quinidine and doxycycline, after which his QTs returned to baseline, and quinidine was stopped. Six hours later, he received the first ART dose per protocol (2.4 mg/kg at 0 h, 12 h, 24 h, and 48 h). His whole body parasitaemia went from 42% to 0.4% within 2 days since ART initiation and showed improved metabolic acidosis. After 18 days, he showed full neurological recovery and was discharged [59].

Thera et al. evaluated the impact of seasonal malaria chemoprevention using four ART–amodiaquine (ART–AQ) doses at one-month intervals in 200 school-aged Malian children. The students were randomized into two groups each with 100 participants, either to receive ART–AQ monthly or as a control with no intervention. The control group had 20 uncomplicated malaria cases, while only 3 cases were reported among those who received ART–AQ, and no severe malaria was recorded [60].

A total of 64% of children who received ART–AQ had abnormal pains, 44% reported headaches, 22% reported dizziness, and nausea and vomiting were had 7% and 6%, respectively. Dizziness was the adverse effect that lasted the longest at 3.8 days. The reduction in the risk of clinical malaria by 86% and its infection prevalence by 67% showed that seasonal malaria chemoprevention is a potential strategy for eliminating seasonal malaria [60].

Varo et al. conducted a study on post-malarial anaemia in Mozambican children under the age of 15, who were treated with ART or quinine between 1 January 2003 and 31 December 2017. A total of 23,523 children were hospitalized during this period, and 9461 were discharged alive, while 62 deaths were confirmed. Of the 62 casualties, 55 were treated with quinine, while only 5 were treated with ART. Of those discharged, 1519 children had known haematocrit values within 7–28 days after being discharged from the hospital, and 1333 were put on quinine, while 154 were treated with ART. A total of 6 deaths and 305 post-malarial cases of anaemia were confirmed on those treated with quinine, while no deaths and 39 post-malarial anaemia cases were reported from the ones treated with ART. The results showed the need for active follow up on children with severe malaria, due to the prevalence of post-malarial anaemia [61].

### 2.3. The Therapeutic Effects of Artesunate on Cancer

ART is known for its efficacy against various cancers, such as liver cancer [17], leukaemia [62], and breast cancer [63]. Apart from the endoperoxide moiety, the heme that cancer cells produce plays an important role in activating ART, producing abundant free radicals that damage cancer cells [16]. The anticancer effects of ART work through affecting various processes and pathways in cancer cells, such as arresting the cell cycle [14], inhibiting angiogenesis [64] and ferroptosis [65], inducing apoptosis [14], inhibiting proliferation [66], and inducing autophagy [67].

Jing et al. reported the ability of ART to enhance the sensitivity of hepatocellular carcinoma (HCC) to sorafenib (Sor), a tyrosine kinase inhibitor used to treat advanced primary liver cancer [68]. Node mice having SK-7721 tumour cells were treated with Sor, ART, or a combination of the two (ART–Sor). Treatment with ART–Sor showed better tumour growth reduction than either ART or Sor alone. In vitro effect of the two showed that the 50% inhibition of SK cells was achieved by 2.77 µM of ART–Sor compared with 5.23 µM Sor alone that was needed for the same effect. For in vitro SM live cancer cells, the inhibition exhibited by 11.43 µM of ART–Sor equalled that of 5.30 µM of Sor alone. ART was found to positively synergize Sor by increasing annexin V^+^ SK cell production and increasing cleaved PARP and caspase-3, thus inducing pro-apoptotic processes. In vivo studies showed that ART–Sor enhanced HCC apoptosis by inhibiting both P13K/AKT/mTOR and MAPK pathways [17].

Mancuso et al. evaluated the in vitro and in vivo effects of ART in leukemic cells (U937 and HL-60). Treatment of U937 and HL-60 cell lines with 1 mM ART initiated a stress response 1 h and 2 h, respectively, after drug treatment. Phosphorylation of eIF2α at 4 h and 6 h in U937 and HL-60 cell lines, respectively, activated activating transcriptional factor 4 (ATF4), central to PERK-governed signalling. At 6 h and 12 h post ART treatment, the transcription factor C/EBP homologous protein (CHOP) was activated in U937 and HL-60 cell lines, respectively. Apoptotic cell percentage increased by 25.9% in U937 and 19.1% in HL-60 cell lines 24 h after ART treatment. The average tumour volume decreased by 52% on the ART-treated tumours [62].

Pirali et al. evaluated the apoptosis-inducing effects of ART in breast cancer cells (4TI and MCF-7), through the inhibition of HSP70 ATPase activity. In vitro analysis showed that incubating HSP70 with 10, 30, and 50 µM ART inhibited carbonic anhydrase unfolding by 9, 19, and 38%, respectively. Inhibition of ATP hydrolysis of HSP70 also followed a concentration-dependent pattern. Inhibition rates of 4TI cells proliferation by different ART concentrations, 1, 5, 10, 25, 50, and 100 µM, were 3.66, 10.73, 17.53, 31.84, 47.56, and 67.92%, respectively, when compared with the control [63].

The same ART concentrations gave proliferation inhibition rates of 3.48, 8.68, 13.49, 24.25, 39.21, and 60.48% against MCF-7 cells. In both 4TI and MCF-7 cells, ART downregulated Bcl-2 and HSP70 expression while it enhanced cleaved caspace-9 expression. An 18 h exposure of the MCF-7 and 4TI cells to ART increased caspase-9 activity in both cell lines; thus, these results showed that ART induces apoptosis in breast cancer cells by inhibiting HSP70 expression [63].

Trimble et al. assessed the efficacy and safety of self-administered ART vaginal inserts in biopsy-confirmed cervical intraepithelial neoplasia 2/3 (CIN2/3) patients by conducting a first-in-human phase I escalation study. A total of 28 patients were included in the study which included four treatment groups, each group receiving 1, 2, or 3 five-day treatment cycles at 0, 2, and 4 weeks, respectively. Participants reported mild adverse events such as dizziness, uterine cramping, urinary tract infection, and headaches [69].

The modified intention-to-treat analysis showed that 19/28 (67.9%) participants showed histologic regression. Of the 19 participants, 9 of them had a clearance of HPV genotypes that were detected at baseline, with 3 of the 9 having viral clearance occurring concurrently with histologic regression. Viral clearance was longer in patients who had one treatment (mean 27.5 weeks, *n* = 9) compared with those who received two or three treatment cycles (mean 16.5 weeks, *n* = 10). The results showed the good tolerance and safety of self-administered vaginal ART inserts in treating CIN2/3 [69].

Fei et al. found that ART inhibited the growth of oesophageal cancer cells (KYSE-150, KYSE-410, and TE-1), with IC_50_ values of 80.2, 75.7, and 55.3 µmol/L, respectively. ART was around 3-fold more cytotoxic on the oesophageal cancer cells compared with an IC_50_ value of 199.6 µmol/L on normal oesophageal cell line HEY-1A. TE-1 cells treated with 5 µM ART and 0–8 Gy X-ray radiation showed a lower clonogenic survival fraction compared with the cells that were exposed to radiation only. ART enhanced radiation-induced apoptosis in TE-1 cells from 4.9 ± 0.5% in the control to 34.1 ± 2.5% and decreased the percentage S phase TE-1 cells population. ART downregulated RAD51, Ku70, cyclin D1, Ku86, and RAD54 protein expression, thereby contributing to delay of deoxyribonucleic acid double-strand break (DNA DSB) damage repair. In vivo studies showed that mice treated with ART + radiation had lower tumour volume and weight compared with those treated with radiation alone. Thus, ART enhances oesophageal cancer cells’ radiosensitivity through inhibition of DNA damage repair [64].

Wang et al. studied the role played by ART in the treatment of several Burkitt’s lymphoma (BL) cell lines. In vitro results showed that ART inhibited DAUDI (IC_50_ = 2.75 ± 0.39 µM) and CA-46 (IC_50_ = 2.73 ± 0.68 µM) cell proliferation and induced their death. Treating the two cell lines with ART increased ROS generation and lipid peroxidation, indicating ferroptosis induction. ART activated the ATF4–CHOP–CHAC1 pathway, and this in turn unregulated CHAC-1 expression, thus enhancing ART-induced ferroptosis in BL cells. In vivo results showed an average tumour volume of 2.82 ± 0.33 cm^3^ in the control group and 1.45 ± 0.16 cm^3^ in the ART-treated group, indicating the ability of ART to inhibit tumour growth. Average subcutaneous tumour weight also decreased in the mice treated with ART compared with the control [15].

Juengel et al. treated renal cell carcinoma (RCC) cell lines (KTCTL-26, A498, 786-O, and Caki-1) with sunitinib to induce sunitinib resistance. Treatment of the cell lines with ART showed significant inhibition of tumour cell growth and proliferation when compared with untreated cells. Ferroptosis was found to be responsible for the inhibition of these therapy-resistant RCC cells. ART treatment caused cell cycle arrest in the G_0_G_1_ phase and changes in the expression of proteins of the Akt/mTOR signalling pathway [66].

Li et al. explored the in vitro and in vivo anticancer effects of a combination of ART and cisplatin on A549 cells. Both ART and cisplatin inhibited A549 cell growth in a dose-dependent manner. CCK-8 assay results showed that treating A549 cells with a combination of ART and cisplatin caused more reduction and induced a distinct decrease in colony formation compared with either of the two alone. The combined treatment showed an increase in apoptotic cells of 29.7% compared with 16.1% for ART and 20.0% for cisplatin [14].

Cell cycle arrest in the G_2_/M phase increased from 16.1% for ART and 17.39% for cisplatin to 35.13% for the combined ART + cisplatin treatment. The combined treatment affected the expression of Bcl-2, Bax, p-P53, caspase-3/7, caspase-9 [70], cyclinB1, P34, and P21 and synergistically regulated the P38/JNK/ERK1/2 MAPK pathway activity. In vivo studies showed that ART has chemo sensitization effects with cisplatin and that the combined treatment had more significant tumour growth inhibition (75.7%) compared with 19.1% and 34.1% for ART and cisplatin, respectively [14].

Xiong et al. prepared PLGA porous microspheres loaded with ART for treating non-small cell lung cancer (NSCLC). The ART-loaded PLGA microspheres had a 90.09% cumulative ART release within 8 days, and the ART was efficiently taken up by the cells. In vitro anti-proliferative effects using A549 cell line showed that the cell viability of the release solutions at days 1, 3, 5, and 8 were 36.92, 19.19, 10.00, and 5.33%, respectively, and that the day 8 solution killed almost all the cells. ART released from the PLGA microspheres inhibited colony formation, induced apoptosis by inhibiting Bcl-2 [70], and arrested cell cycle at G_2_/M phase with ratios of 31.52, 38.17, and 40.94% from ART contained in release solutions taken at day 1, 3, and 5, respectively. The ART from the release solution collected at day 5 completely inhibited cell migration, with the largest wound area of 97.82%, suggesting that the ART-loaded PLGA microspheres can prevent migration and invasive behaviour of cancer cells [71].

Wei et al. investigated the anticancer effects of ART on glioma cells (SK-N-SH, U87, U251, and U138). ART induced apoptosis and impaired motility of U87 cells and inhibited 3-hydroxy-3-methylglutaryl coenzyme A reductase (HMGCR) expression, which resulted in inhibition of the anchorage-independent growth of U87 and U251 cells through negative mevalonate pathway regulation. In vivo studies showed that ART administration improved survival of mice with U251 cells, decreased the number of metastatic foci formed in the lung, and led to near-diminishing of HMGCR expression in the lungs of mice [72].

ART lowered mRNA levels of HMGCR in U87 and U251 cells compared with their counterpart cells. Moreover, ART inhibited SREBP2 binding to the HMGCR promoter, resulting in less nuclear-localization of SREBP2 in ART-treated U87 cells compared with untreated ones. ART showed its ability in disruption of SREBP2–P53 interaction, induction of P21 expression, and promotion of senescence in glioma cells [72].

Kumar et al. evaluated the ability of ART in suppressing inflammation and oxidative stress in colorectal cancer. ART lowered the histology score value to 7.00 ± 0.26, which was close to that of 3.67 ± 0.21 in the control group. ART treatment normalized oxidative stress markers such as GSH and superoxide dismutase (SOD) activity and produced a dose-dependent reduction in the level of inflammation markers, with effects the same as those of aspirin. ART reduced IR score of markers such as COX-2, nitric oxide (iNO)S, NF-κB (p65), and IL-1β, and the IC_50_ value of ART against COX-2 enzyme activity was 743.34 μg/mL, compared with 592.54 μg/mL of aspirin. Thus, ART has the potential in reducing colon cancer, similar to aspirin [73].

Zhang et al. studied the underlying relationships among ART autophagy, CD155 (a type 1 transmembrane glycoprotein whose overexpression plays a critical role in cell proliferation and migration) [74], and their roles in uterine corpus endometrial carcinoma (UCEC) progression in vitro. ART exhibited potent anticancer activity by inhibiting migration and proliferation and by promoting apoptosis of UCEC cells [67].

ART was thought to trigger biological processes such as response to oxidative stress and steroid hormone and liposaccharide and ameboidal-type cell migration. mTOR signalling pathway was noted as the critical regulatory axis in the autophagy of UCEC cells. CD155 was found to be upregulated in UCEC cells and associated with the PI3K/AKT-mTOR and mTORC1 signalling pathways, indicating its correlations with autophagy. The upregulation of CD155 in UCEC cells by ART was partially through ATG5, and this promoted cytotoxicity of NK92 cells through interactions between CD155 and CD226 and TIGIT, its receptors [67].

Zhou et al. reported the efficacy of ART on human bladder cancer cells. Apart from inhibiting cell proliferation and migration, ART also induced both caspase- and autophagy-dependent apoptosis in T24 and EJ cells. ART activated the AMPK–mTOR–ULK1 pathway, which plays a critical role in autophagy activation. Treatment of EJ and T24 cells with ART showed significant upregulation of ROS level in a dose-dependent manner and ROS upregulation-initiated AMPK–mTOR–ULK1 axis [16].

### 2.4. Artesunate Efficacy in Viral Infections, Skin Diseases, and Diabetes

Recent research has demonstrated the broad antiviral potency of ART [75]. These viruses include herpes, human cytomegalovirus (HCMV) [10], rabies [76], etc. ART has been shown to have some anti-fibrosis efficacy [77] and can inhibit dermatitis due to its anti-inflammatory properties [78]. The activity of ART depends on its ability to regulate signalling pathways such as the P13K/AKT and P13K/AKT/mTOR [79]. ART reduces the concentration of glucose in the blood, and this is important in preventing the onset and progression of diabetes [80].

Since the arrival of the coronavirus disease of 2019 (COVID-19), researchers have found themselves in a race against time to find effective treatments [81]. Zhou reported ART to be the most potent of the artemisinin against SARS-CoV-2 infection of Vero E6, human lung cancer, and human hepatoma, in the EC_50_ range of 7–12 µg/mL [82]. Gendrot et al. evaluated the in vitro antiviral activity of fixed ACT concentrations against a clinically isolated SARS-CoV-2 strain (IHUMI-3) in Vero E6 cells. Combinations such as artemether–lumefantrine, ART–amodiaquine, ART–piperaquine, and ART–pyronaridine exhibited antiviral inhibition in the range 27.1% to 34.1% (Table 4). The combination of mefloquine and ART exhibited the highest antiviral activity, with replication inhibition of 72.1 ± 18.3% at the expected maximum blood concentration. This shows that ACTs, particularly mefloquine–ART can be a potential intervention against SARS-CoV-2 [83].

The trimeric derivative of ART, TF27, is known for its antiviral activity against several viruses [84]. Jacquet et al. conjugated three ART molecules to form the compound TF27 and evaluated their in vitro and ex vivo antiviral activity against human cytomegalovirus (HCMV). When compared with ART, TF27 showed higher in vitro efficacy against HCMV replication against AD169 strain, endotheliotropic strains VHL/E and TB40/E, and clinical strain S^*^. The EC_50_ values of TF27 were in the nanomolar range, while those of ART were in the micromolar range [85].

**Figure 3:**
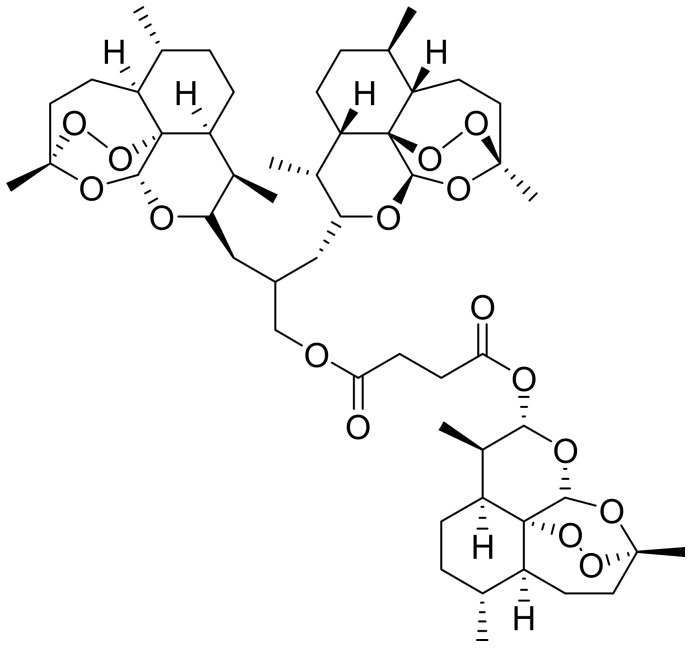
Chemical structure of TF27.

TF27 (Figure 3) exhibited a minor impact on cellular death on HEF cells, endothelial cells (HUVEC), and epithelial cells (ARPE-19) at both EC_50_ and EC_90_, and the maximum cytotoxicity was at 11.84 ± 11.90% at TF27 EC_90_. HCMV strains AD169, TB40/E, and VHL/E infected placental villi, with AD169 strain reaching a peak of 2.6 × 10^6^ viral copies per million cells after incubation for 10 days and was stable at day 13. Both ART and TF27 significantly decreased viral load in the first-trimester placenta explants, with TF27 showing EC_50_ values 200-fold lower than that of ART, making it a potential candidate for congenital HCMV treatment [85].

Wild et al. reported on the prophylactic efficacy of TF27 against murine and human cytomegalovirus. TF27 showed better HCMV replication (EC_50_ = 0.04 ± 0.01 μM) than ART (EC_50_ = 5.41 ± 0.61 μM). Both ART and TF27 showed sustained efficacy after withdrawal by reducing viral replication by at least 50%. In vivo studies showed that orally treating virus-infected mice with TF27 significantly reduced viral load in spleen samples, where the highest viral load was detected, compared with valganciclovir-treated control animals [75].

TF27 is known to interact with mitochondria in HCMV-infected cells by altering its morphology and downregulating mitochondrial proteins, leading to loss of mitochondrial function [19]. The in vivo studies showed no compound-related side effects. Thus, TF27 is an effective inhibitor of cytomegaloviral (CMV) replication and can be used for prophylaxis against CMV infections [75].

Shenoy et al. reported the efficacy of ART on treating CMV reactivations in allogeneic haematopoietic stem cell transplant (ASCT) recipients intolerant or not suitable for ganciclovir therapy. The study included 117 patients who underwent ASCT, and 78 episodes of CMV reactivation were recorded in 68 patients (58%). A total of 25 patients for 27 out of the 78 episodes received ART, 6, 13, and 8 episodes as first-, second-, and third-line agents, respectively [10].

At the start of ART treatment, the median CMV viral load was 1.6 × 10^3^ copies per mL. A total of 5 (19%) of the 27 CMV episodes were cleared with ART. However, ART effectively controlled CMV proliferation in 20 (74%) of the 27 episodes. ART failed in 6 (22%) of the 27 episodes. A total of 22 patients showed no major side effects from ART treatment while 3 patients developed haemolysis thought to be ART-related. A failure rate of less than 25% and complete clearance at 19% showed that ART is effective against CMV and can be used in patients cytopenic and intolerant to ganciclovir [10].

Luo et al. determined whether ART could enhance the immune response of the rabies vaccine when used as an adjuvant. Treatment of mice for 15 days at a dose of 5 mg/kg neither caused side effects nor weight loss. Immunizing mice with inactivated CVS-11 (rabies strain) + ART caused higher virus-neutralized antibody (VNA) level in the peripheral blood, and ART significantly enhanced VNA induction in mice immunized with inactivated rabies strain rHEP-dG. Since mice treated with rHEP-dG + ART had a higher survival rate compared with those treated with rHEP-dG + PBS, it showed that ART can enhance the protective effect of the rabies vaccine [76].

Kumaran et al. reported a case whereby a neonate presented chromosomally integrated human herpes virus 6A (ciHHV-6) myocarditis within 30 min of birth. No family history could be traced to be a cause of the condition. Viral myocarditis signs included sinus rhythm with low-voltage complexes and elevated troponin-I, brain natriuretic peptide, and creatinine phosphokinase. Administration of inotropes (drugs used to improve basic functions of the cardiovascular system) caused minimal improvement [86]. The patient was then administered ART at a dose of 5 mg/kg/× 10 days, followed by an oral dose of 5 mg/kg/dose twice a day ×10 days on day 4. This resulted in the patient being waned off respiratory support, starting on direct breastfeeds, and being discharged 12 days after birth. After the completion of ART, cardiac contractility increased from 40% on day 2 to 55% on day 34 and was normalized by day 63 [11].

Huang et al. studied the therapeutic efficacy of ART on imiquimod (IMQ)-induced psoriasis-like dermatitis in mice. ART delayed initiation and progression of dermatitis and notably ameliorated IMQ-psoriasis severity compared with the IMQ model group. ART reduced keratinocyte layers, especially epidermis hyperplasia. Studies on the anti-hyperplasia effect of ART on keratinocytes showed that ART reduced the number of Ki-67 positive basal layer cells from 19.1 ± 2.3 to 12.5 ± 1.6, meaning ART could reduce the proliferation of the epidermis during psoriasis lesion development. ART treatment inhibited the proliferation of the spleen, draining lymph nodes cells, and reduced the population of γδ T cells in draining lymph nodes cells [87].

Larson et al. investigated the effects of ART on myofibroblast formation on CRL-2097 human dermal fibroblasts. ART reduced α-SMA basal levels, antagonised TGF-β-mediated expression of α-SMA, and decreased deposition of collagen I and III. ART notably downregulated the expression of several myofibroblast-associated and pro-fibrotic genes. *MMP1* and *MMP3*, two genes associated with extracellular matrix proteins degradation, had their transcript expression upregulated. The expression of *CDKN1A* and *CDKN2A*, the genes responsible for encoding cell cycle inhibitors, was upregulated. ART induced fibroblasts cell death, which was likely to proceed via apoptosis [88].

Shen et al. tested the efficacy of ART against schistosomiasis-induced liver fibrosis and its mechanism of activity in a mouse model. Comparing the mouse liver from the control, infection, and ART intervention groups showed that the area of fibrosis and granuloma observed in the infection group was decreased in the ART intervention one. Fibrosis-related genes found in the liver such as Col1a1, Col3a1, and α-SMA were significantly downregulated in the ART intervention compared with the infection group. ART increased the apoptosis of LX-2 cells by 17.2% at 200 µM and reduced Hepatic stellate cells (HSCs) viability in a time- and dose-dependent manner. ART inhibited mitochondrial function by decreasing OCR, ATP production, basal and maximal respiration, and spare respiratory capacity. ART decreased the expression of respiratory chain proteins in complex III subunit UQCRC2 and complex I subunit NDUFB8, resulting in decreased mitochondrial activity [89].

Wan et al. investigated the effect of ART on inhibiting fibroblast proliferation via autophagy-mediated p53/p21^waf1/cip1^ pathway and its ability to reduce the formation of epidural fibrosis post lumbar laminectomy. ART inhibited fibroblast growth in a dose- and time-dependent manner, arrested the progression of the cell cycle in the G2/M phase, and decreased the expression of PCNA and cyclin D1, which are cell proliferation markers. ART induced cellular autophagy cascade, as indicated by the increase in the expression of autophagic flux-related proteins [90]. Treatment of fibroblasts with ART increased the expression levels of p53/p21^waf1/cip1^ proteins. Intragastric ART administration reduced fibrous adhesion tissues in a dose-dependent manner and could reduce collagen synthesis in epidural fibrosis tissue [90].

Bai et al. investigated the effectiveness of ART in attenuating 2,4-dinitrichlorobenzene (DNCB)-induced atopic dermatitis (AD). The mice were divided into four groups: untreated, AD-induced mice (DNCB group), and AD-induced treated with either 5 mg/kg ART (AS-L group) or 10 mg/kg ART (AS-H group). DNCB induced the development of oedema, scaling, haemorrhage, and dryness in mice, and these symptoms were relieved following ART treatment [78].

The weight of the spleen and lymph nodes, which were remarkably high in the DNCB group, were significantly reduced upon treatment with ART. The AS-L and AS-H treatment attenuated DNCB-induced AD-like skin inflammation and decreased the number of skin mast cells. ART treatment decreased TNF-α levels, suppressed the IL-4 and IL-5 mRNA expression, and reversed the DNCB-induced change of Th17-related cytokine expression, showing its ability to attenuate DNCB-induced AD in mice [78].

Kong et al. explored the role played by ART on hepatic stellate cells (HSCs) ferroptosis and evaluated its molecular and cellular mechanisms. ART significantly decreased four indicators of liver fibrosis in vivo. ART-treated activated HSCs had increased lipid ROS, Fe^2+^, and Ptgs2 mRNA levels and decreased GSH content, showing ART-induced ferroptosis. In vitro studies showed ART-induced HSCs death and suppressed viability and resulted in smaller, broken, and crumpled mitochondria, characteristic of ferroptosis [77].

Treatment of activated HSCs with ART, ferroptosis specific inhibitor iron chelator deferoxamine (DFO), or co-treatment for 24 h showed that blocking ferroptosis abolished ART-induced antifibrosis efficacy in vitro. ART promoted autophagic flux appearance of ferritinophagy-related genes and markers. ART-induced ferroptosis and anti-fibrosis function were inhibited by co-treating HSCs with lysosomal lumen alkalizer, chloroquine. The results showed that ART can alleviate liver fibrosis through the regulation of ferroptosis signalling pathway [77].

Wan et al. explored how ART affects intraarticular fibrosis progression post knee surgery and the underlying molecular mechanism. ART treatment of fibroblasts showed that it decreased DNA synthesis, arrested cell cycle, and decreased cell proliferation markers (PCNA and cyclin D) expression. ART increased protein level of Atg5 and decreased LC3-II/LC3-I ration while significantly increasing p62 expression level in fibroblasts with LV–Beclin-1–shRNA. Treating fibroblasts with ART for 24 h reduced phosphorylation levels of both p70S6K and mTOR while increasing phosphorylation level of AMPK, showing that ART induced autophagy by inhibiting mTOR signalling through the AMPK/mTOR and P13K/AKT/mTOR pathways. In vivo results showed that ART induced autophagy activation and reduced surgery-induced knee arthrofibrosis [79].

Li et al. studied the role and mechanism of ART in nonobese diabetic (NOD) mice using a mouse model of type 1 diabetes. A total of 62.5% of the mice treated with DMSO became diabetic, while only 25% of those treated with ART became diabetic. Although ART administration lowered the degree of insulitis, it did not revert the disease in NOD mice. ART administration increased the frequency of T_h_2 cells and decreased T_h_1 cells in the spleen and pancreatic lymph nodes. Changes in cytokine production in CD8^+^ T cells were observed in the spleen and pancreatic lymph nodes [12].

TNF-α and IL-6 levels decreased, IFN-_ϒ_–secreting T cells frequency were reduced, and the proportion of IL-4-producing cells and T_reg_ cells increased post ART administration. In vitro studies showed that ART increased both Ins1 and Ins2 levels in the significantly increased β-cell mass, increased molecules responsible for maintaining functional maturity of β cells, and decreased the endocrine progenitor marker *Ngn3* expression [12].

Ackermann et al. determined whether treating mice with ART for a long time induced α-to-β cell transdifferentiation, as was put forward by Ben-Othman et al. and Li et al. in 2017. Although the mice that were treated with ART for 3 months showed faster clearance of glucose than those treated with DMSO, 2 hr ending and fasting glucose levels did not differ between the two studied groups. Plasma insulin concentrations were the same in the two groups at fasting or 30 min during intraperitoneal glucose tolerance tests. ART-treated mice had lower body weight at the end of the 6 months of study [91].

The presence of insulin and YFP co-expressing cells and the same fractions of insulin^+^/YFP^+^ cells between the two groups showed no evidence of α-to-β cell transdifferentiation induced by ART. No difference in islet number, islet size distribution, islet area, β cell area per islet, or number of YFP^+^ cells per islet was noticed between the ART-treated and the control groups. The findings showed that ART does not induce pancreatic α-to-β cell transdifferentiation in vivo [91].

Sun et al. evaluated the in vitro effect and molecular mechanism of ART on diabetic nephropathy (DN). Treating rat mesangial cell line HBZY-1 cells with ART inhibited high glucose-induced proliferation. High glucose stimulation resulted in the induction of mRNA levels of IL-6, IL-1β, and TNF-α and increased inflammatory cytokines production and treating the cells with ART attenuated the induction and inhibited the production of high glucose-induced inflammatory cytokines. ART inhibited the increase in ROS and MDA levels, attenuated SOD activity inhibited by high glucose, and decreased the expression of extracellular matrix proteins such as collagen IV, laminin, and fibronectin. The NF-κB, TLR4/MyD88, and NLRP3 inflammasome pathways were all involved by ART in high glucose-induced HBZY-1 cells and formed a signalling axis which when inhibited, reversed the effect of high glucose on HBZY-1 cells [92].

Alagbonsi et al. studied the gender- and duration-based mechanism of the ART effect of glucose homeostasis by evaluating the enzymes and hormones involved. A total of 50 albino rats (25 male and 25 female) were separated into five groups that received different ART concentrations for day 15. ART reduced the concentration of plasma glucose better in female rats than in males. Liver glycogen was increased just after ART administration but normalized with time. After 15 days, only the male rats had increased liver glucose-6-phosphate concentration, whereas liver glucose-6-phosphatase concentration decreased with no difference in both sexes [80].

Only male rats treated with low ART for 5 days had increased insulin. Cortisol concentration increased in males and decreased in females. Male rats that received low ART for 5 days had increased oestrogen, whereas female rats treated with high ART dose for 15 days had it reduced. The results showed that ART reduces plasma glucose through reduction in glucagon concentration and inhibition of glucose-6-phosphatase in both sexes and that only in male rats did an increase in insulin concentration contribute to plasma glucose reduction by ART [80].

### 2.5. Other Recent Reports on the Biological Activities of ART

Shang et al. investigated the effect of ART on sepsis-induced immunosuppression using a caecal ligation and puncture (CLP)-induced mouse model. ART decreased mortality rate from 85% to 50% in CLP mice with *Pseudomonas aeruginosa*. ART treatment significantly raised TNF-α, IL-1β, and IL-6 levels and decreased bacterial load in CLP mice with *Pseudomonas aeruginosa*. In vitro and in vivo results showed that ART enhanced the release of pro-inflammatory cytokine and promoted bacterial clearance, thereby reversing sepsis-induced immunosuppression [93].

Mechanism of action studies showed that ART interacted with vitamin D receptors by inhibiting its biological function through preventing its nuclear translocation, thereby affecting the transcription of its target gene Atg16l1. ART inhibited vitamin D receptors from interacting with NF-κB p65 in LPS-tolerant macrophages and activated NF-κB p65 target genes transcription. The results showed a novel way of using ART to reverse sepsis-induced immunosuppression [93].

Zhang et al. evaluated the ability of ART postconditioning in decreasing lipopolysaccharide (LPS)-induced sepsis-induced acute lung injury (ALI) in rats and the mechanisms involved. Histopathological evaluation of lung samples from the LPS group revealed acute alveolar injury, which was treated with 15 mg/kg ART. TNF-α and IL-6 in BALF and lung W/D ratio, which were elevated in LPS group, were significantly reduced by ART treatment. ART treatment reduced lung myeloperoxidas (MPO) activity and malondialdehyde (MDA), glutathione peroxidase (GSH-Px), and superoxide dismutase (SOD) levels, which are the indicators of oxidative stress injury [23].

Immunofluorescence analysis showed enhancement of the mTOR/AKT/PI3K axis intensity and attenuation of IL-6 and TNF-α levels relative to the LPS group. ART administration attenuated caspase-3 levels and enhanced protein levels of p-Akt/Akt, p-mTOR/mTOR, and PI3K/β-actin when compared with the LPS group. TUNEL analysis showed decreased levels of the apoptotic index, while treatment with LY294002 inhibited the function of ART [23].

Zhan et al. evaluated the biological efficacy of ART in attenuating Sjogren’s syndrome (SS)-like symptoms in vivo and explored the in vitro mechanism involved. ART-treated NOD/Ltj mice had a higher salivary flow rate, showed decreased lymphocytic foci numbers, and alleviated lymphocyte infiltration compared with untreated mice. ART-treated NOD/Ltj mice had lower levels of RF and IgG and significantly fewer B220 + B lymphocytes in the foci than the untreated mice. ART inhibited B cell-activating factor (BAFF)-induced survival of B cells by increasing the apoptotic ratio through downregulation of the NF-κB signalling pathway. ART affected protein levels of TRAF6 in Raji cells and enhanced the ubiquitination level of TRAF6, thereby causing TRAF6 degradation and NF-κB inhibition. The results showed the ability of ART to determine the survival and proliferation of B cells by ameliorating murine SS-like symptoms and regulating TRAF6–NF-κB signalling [94].

He et al. evaluated the ability of ART in inhibiting atherosclerosis plaque formation and the mechanism involved. Incubation of vascular smooth muscle cells (VSMCs) with 100 µM ART for 24 h increased the expression levels of lipoprotein lipase (LPL) protein and mRNA. ART increased the protein and mRNA levels of TCF7L2, thus enhancing LPL promoter activity. ART increased the nuclear levels of NRF2, upregulated KLF2 expression, and restrained reduction in NRF2 nuclear levels, thereby increasing LPL expression in VSMCs. ART caused no liver or kidney damage in the ApoE^±^ mice used for the study. ART treatment caused a decrease in lipid deposition and increased LPL expression in VSMCs and VSMC count in atherosclerotic plaques. The results showed that ART can inhibit atherosclerosis through upregulation of VSMCs-derived LPL expression via the KLF2/NRF2/TCF7L2 pathway [21].

Su et al. evaluated whether ART attenuates bone erosion during rheumatoid arthritis (RA) progression. ART significantly reduced the total areas and number of F-actin rings and TRAP-positive multinucleated cells and inhibited RANKL-induced pit-formation, suggesting its ability to inhibit osteoporosis. ART inhibited the generation of ROS during osteoclast differentiation and osteoporosis. ART treatment caused an upregulation of NRF2 in the nuclei, which was caused by p62 accumulation [22].

Impairment of NRF2 activation was noted in p62 knockdown cells, together with enhancement of CTSK, TRAP, MMP-9, and NFATc1 expression, suggesting the resistance of p62 knockdown cells to the antiarthritic effects of ART in osteoclastogenesis. Collagen-induced arthritis (CIA) rat model not only showed that ART protected against oxidative damage but also attenuated arthritic bone destruction by increasing the activity of SOD, an antioxidant enzyme, in the inflamed ankle joints [22].

Zeng et al. studied to validate their speculation that ART can inhibit DKK1 expression by upregulating miR-34, thereby activating Wnt signalling resulting in osteogenic differentiation of human bone marrow mesenchymal stem cells (hBMSCs). Treating hBMSCs with 2.5–10 μM ART increased alkaline phosphatase (ALP) activity, Alizarin Red S (ARS) staining, and the mRNA and protein expression of osteocalcin (OCN), Runx2, and osteopontin (OPN), suggesting the ability of ART in promoting osteogenesis. ART treatment activated the Wnt pathway by downregulating DKK1 and upregulating β-catenin, cyclin D1, and c-myc, but overexpression of DKK1 proved to inhibit ART osteogenesis promotion [95].

ART treatment enhanced miR-34a expression, and low levels of miR-34a decreased ALP activity and ARS staining of hBMSCs. Treating hBMSCs with an miR-34a inhibitor increased DKK1 levels and decreased protein expression of β-catenin, cyclin D1, and c-myc. Bioinformatics studies showed the existence of a direct binding site between DKK1 and miR-34a mimic. Thus, ART can promote osteoblast differentiation through the miR-34a/DKK1/Wnt pathway [95].

Dang et al. investigated how ART affects follicular helper T (Tfh) and T follicular regulatory (Tfr) cells on lupus-prone MRL/*lpr* and the mechanism involved. Systemic lupus erythematosus (SLE) is characterized by high proteinuria, serum creatinine, and urea nitrogen levels. Treatment with 2.5 and 5 mg/kg ART reduced the mice mortality rate, inhibited proteinuria increase and reduced serum creatinine and urea nitrogen levels. ART alleviated renal damage and decreased levels of anti-dsDNA IgG but not IgM antibodies in kidneys [96].

Pro-inflammatory cytokines such as IFN-γ, IL-6, and IL-21, which are elevated in SLE patients, were reduced significantly upon treatment with ART. ART inhibited Tfh production and increased the Tfr to Tfh cells ratio. An amount of 5 mg/kg ART significantly decreased the expression of p-Jak2/Jak2 and p-Stat3/Stat3 better than the 2.5 mg/kg treatment, suggesting that ART has therapeutic effects on lupus-prone MRL/*lpr* mice and involves Tfh cells and JAK–STAT signalling pathways [96].

Mota et al. verified the specific chromosome damage and changes in oxidative–nitrosative stress markers and apoptosis triggered by exposure to ART in human peripheral blood lymphocytes. Incubation of cultured lymphocytes for 24 h with ART significantly increased C^+^ MN and C^–^ MN levels, showing its ability to induce aneugenic and clastogenic events [65].

Determination of superoxide anion (O_2_^−^) and nitric oxide (NO) levels as the major ROS and nitrogen reactive species revealed that 1 h incubation with 1 μg/mL ART increased both O_2_^−^ and total NO (about 16%) compared with the control, and only NO levels changed when the ART concentration was raised to 2 μg/mL. Exposing cultured human lymphocytes to 2 μg/mL ART for 24 h upregulated the amount of caspases 8 and 9 and cytochrome c in comparison with the control. Thus, ART induces changes in oxidative–nitrosative levels of human lymphocytes, causing apoptosis and aneugenic and clastogenic effects [65].

Wang et al. investigated the immunomodulatory effect of ART on hydrocortisone (HC)-induced immunosuppression. The HC mice model was established by intramuscular administration of 20 mg/kg HC once per day for 5 days. HC mice administered with different doses of *E. coli* showed very low ability to clear the bacteria and worse ability to release TNF-α. Although no blood clearance was noticed by administering 20 mg/kg ART from day 6–10, TNF-α and IL-6 levels increased, and the bacterial load significantly decreased upon treating the HC mice with ART twice a day and continuing 2 days after HC administration was stopped. ART (2.5 μg/mL) increased TNF-α and IL-6 mRNA expression and their release from HC cells. ART also inhibited glucocorticoid-induced leucine zipper (GILZ) mRNA expression and increased both TLR4 and NF-κB expressions; thus, ART poses immunomodulatory effects [97].

Ghoneim et al. investigated the potential beneficial effects of ART and/or rapamycin (Rapa) against hepatic I/R injury and the mechanism involved. Treating hepatic I/R injured rats with ART and/or Rapa hampered caspase-1/caspase-11 NLRP3 inflammasome. ART normalized all the pyroptosis components, and a combination of ART and Rapa improved all inflammasome markers. ART showed better anti-inflammatory ability by inhibiting *HMGB1/RAGE* and *TLR4/MyD88/TRAF6* signal compared with either Rapa alone or ART + Rapa. ART lowered the levels of pro-inflammatory cytokines TNF-α and IL-6. ART administration boosted antioxidant defences and ebbed markers of neutrophil infiltration and lipid peroxidation better than Rapa [98].

Treating hepatic I/R injured rats corrected the Bcl-2/Bax imbalance with either Rapa alone or ART + Rapa. Histopathological results showed that ART lowered serum levels of aspartate aminotransferase, alanine aminotransferase, and lactate dehydrogenase and that it reduced liver injury to grade 1 (same as ART +Rapa), whereas Rapa had a lesser improvement to grade 2. Thus, ART can abate functional and structural I/R-induced hepatic abnormalities [98].

Pan et al. investigated how ART inhibits resistance nodulation division (RND) pumps in *E. coli* and the mechanism involved using MarA, a regulator of RNDs. ART (8000 μg/mL) enhanced antibacterial effects of β-lactams against *E. coli* ATCC35218. ART downregulated the mRNA expression levels of soxS, marA, and rob. ART significantly downregulated mRNA expression levels of RND pumps and increased antibiotic accumulation within ATCC35218. The use of *E. coli* lacking marA showed that just ART lost its antibacterial sensitization; thus, marA, soxS, and rob play important roles in bacterial sensitization of ART. Deleting marA led to ART losing its inhibitory effect on RND pumps. ART was found to bind to MarA central cavity using the K62 and R59 residues, altering its charge distribution, thus interrupting its self-transcriptional activation [99].

Feng and Qiu investigated how ART affects chondrocyte proliferation, apoptosis, and autophagy in the rheumatoid arthritis (RA) rat model through the PI3K/AKT/mTOR signalling pathway. Rats in the RA group had higher white blood cells and platelet count compared with the ones in the ART group, showing the ability of ART to alleviate inflammation. ART exhibited no hepatotoxicity for rats with RA. The ART group had lower mRNA and protein expressions of AKT, P13K, Bcl-xl, Bcl-2, and mTOR of cartilage tissues than the RA group but higher expressions of LC3-II, Bax, LC3-I, and Becline-1 of cartilage tissues and chondrocytes. Chondrocyte proliferation examination (CPE) revealed that ART negatively regulated CPE of RA rats through the PI3K/AKT/mTOR signalling pathway. ART arrested cell cycle at G0/G1 phase and promoted chondrocyte apoptosis and autophagy [100].

Zhang et al. examined the effectiveness of ART combined with fractional CO_2_ laser in a hypertrophic scar model. The combination of ART reduced the size and thickness of hypertrophic scar samples, decreased extra and irregular fibroblasts, and suppressed scar thickness of numerical densities of fibroblasts on area and area density of collagen fibres on the area. The combination of ART and CO_2_ laser suppressed better the contents of type I collagen and the ratio of Col-I and III. Both BMP-7 and Fas expression were enhanced by combining ART with fractional CO_2_ laser. The results showed that ART and fractional CO_2_ laser can be used to treat hypertrophic scars [101].

## 3. Conclusions

Although ART is mainly known as an antimalarial drug, it also exhibits anticancer, antidiabetic, antiviral, anti-inflammatory activities, etc. Its mechanisms of action in treating malaria involve ROS generation, heme polymerization inhibition, and destabilization of parasite membrane. ART can be used alone or in combination with other therapeutic agents for improved biological efficacy. However, haemolytic anaemia has been reported to be prevalent in patients treated with ART for severe malaria, having some other immunity weaknesses.

ART efficacy against cancer cells is due to ferroptosis, arrest of certain phases of the cell cycle, and inhibition of both the P13K/AKT/mTOR and MAPK pathways. ART inhibits ROS generation, which can cause damage to the basic building blocks of the cell, including DNA, protein, and lipids. ART showed comparable inhibition with aspirin (IC_50_ = 743.34 and 592.54 μg/mL for ART and aspirin, respectively) against COX-2 enzyme activity, thus showing potency in colon cancer reduction. ART also showed activity against viruses such as HCMV and ciHHV-6. ART ability to inhibit atherosclerosis, attenuate arthritic bone destruction, promote osteoblast differentiation, and induce changes in oxidative-nitrosative levels of human lymphocytes causing apoptosis and aneugenic and clastogenic effects have been reported. Most of the studies on ART were performed at preclinical phases. The biological efficacy of ART indicates that there is a pressing need for more studies to fully understand its mode of action.

## Figures and Tables

**Table 1:** In vivo antiplasmodial activity of 4CE, ART, and their combination against *P. yoelli nigeriensis*-infected Swiss albino mice.

Dose (mg/kg)	Chemo-Suppression (%) on Day 8 After Suppression	Mean Survival Time (Days)
Vehicle control	0.0 ± 0.0	8.7
9.5 (ED50) ART	55 ± 0.37	14.01
88 (ED50) 4CE	47 ± 0.91	9.8
ART + 4CE	91.4 ± 0.64	16.3
100 ART	99.2 ± 0.05	˃28

Results are mean percentages of *n* = 5.

**Table 2:** Pharmacokinetic parameters of free ART and ART–HEP nanocapsules.

Parameters (Unit)	Free ART	ART–HEP Nanocapsules
C_max_ (µg/mL)	14.13	18.12
MRT_0-t_ (h)	2.44	9.39
CL (L/h/kg)	0.19	0.08
*Ke*	0.49	0.10
t_1/2_ (h)	1.39	4.51

C_max_, peak plasma concentration; MRT, minimum residence time; CL, plasma clearance; *Ke*, elimination rate constant; t_1/2_, elimination half-life.

**Table 3:** PCR-uncorrected treatment outcome among the patients treated with ART + AQ.

Classification	Number (%)	95% CI (%)
Early treatment failure	0	0–3.7
Late clinical failure	3 (3%)	0.6–8.6
Late parasitological failure	1 (1%)	0.0–5.5
Adequate clinical and parasitological response	95 (96%)	90.0–98.9
The cumulative success rate after survival analysis	96.0%	89.7–98.5

**Table 4:** Estimation of the percentage (%) of inhibition of the SARS-CoV-2 replication by fixed-doses of ACT (1× corresponds to expected maximum blood concentration (C_max_) for each ACT drug at doses commonly administered in malaria treatment).

	Inhibition %					
	At 2× Plasma C_max_		At 1× Plasma C_max_		At 0.5× Plasma C_max_	
Combination	Conc.	Mean ± SD	Conc.	Mean ± SD	Conc.	Mean ± SD
Mefloquine–ART	8.3−5	99.6 ± 0.7	4.1−2.5	72.1 ± 18.3	2.0−1.25	30.9 ± 14.1
Amodiaquine–ART	4.0–5.0	85.8 ± 9.9	2.0–2.5	32.3 ± 9.9	1.0–1.25	17.2± 6.5
Pyronaridine–ART	0.5–1.0	38.2 ± 5.7	0.25–0.5	34.1 ± 7.1	0.12–0.25	16.3± 5.0
Lumefantrine–ART	33.0−2.0	37.7 ± 3.4	16.5−0.5	27.1 ± 6.0	8.2−0.25	12.3± 4.4
Piperaquine–ART	1.0–3.1	29.7 ± 16.8	0.5–1.5	29.3 ± 4.6	0.25–0.75	14.0 ± 4.2

Conc. = concentrations (µM); SD = standard deviation.

## Data Availability

Not applicable.

## Abbreviations

ART	artesunate
DIBAL	diisobutylaluminum hydride
RBCs	red blood cells
ROS	reactive oxygen species
NLCs	nanostructured lipid carriers
SMA	severe malaria anaemia
Hb	haemoglobin
PMIF	plasmodium macrophage migration inhibitory factor
ED50	effective dose 50
IC_50_	half-maximal inhibitory concentration
C_max_	maximum serum concentration
Qpcr	quantitative polymerase chain reaction
AQ	amodiaquine
HCC	hepatocellular carcinoma
HPV	human papillomavirus
DNA	deoxyribonucleic acid
RCC	renal cell carcinoma
RNA	ribonucleic acid
HCMV	renal cell carcinoma
ACT	artesunate combination therapy
AD	atopic dermatitis
HSCs	hepatic stellate cells
DMSO	dimethyl sulfoxide
MDA	3,4-methylenedioxyamphetamine
SOD	superoxide dismutase
LPS	liposaccharide
LPL	lipoprotein lipase
ATP	adenosine triphosphate
VSMCs	vascular smooth muscle cells
ALP	alkaline phosphatase
RA	rheumatoid arthritis
